# There is no ‘one size fits all’ model of care for patients with behavioural and psychological symptoms of dementia in hospital

**DOI:** 10.1111/ajag.70063

**Published:** 2025-07-04

**Authors:** Frederick A. Graham, Emily H. Gordon

**Affiliations:** ^1^ Department of General and Geriatric Medicine Princess Alexandra Hospital Brisbane Queensland Australia; ^2^ Australian Frailty Network The University of Queensland Brisbane Queensland Australia; ^3^ School of Nursing Queensland University of Technology Brisbane Queensland Australia

Approximately one in four older patients in hospital are living with dementia and up to two thirds of these patients experience behavioural and psychological symptoms of dementia (BPSD) during their hospital stay.^1^ Behavioural and psychological symptoms of dementia, such as agitation, aggression, resistance to care, sleep disturbance and wandering develop from disease‐induced vulnerabilities to a variety of internal (physiological and psychological) and external (social and physical) stressors.^2^ In hospital, multiple factors, including acute illness (with or without delirium), unmet needs, unfamiliar and complex patient–carer relationships and physical environments, may trigger new or exacerbate preexisting BPSD. Despite BPSD being highly prevalent and associated with poor patient outcomes and occupational violence, it remains a relatively under‐studied topic.

Pajaro, To and Whitehead,^3^ in the *Australasian Journal on Ageing*, make a valuable contribution to the evidence base regarding BPSD in hospital. Their 1‐year retrospective evaluation of admissions from a residential aged care facility (RACF) to a tertiary hospital's acute geriatrics multidisciplinary team identified 608 patients with dementia, 82 of whom presented to hospital due to BPSD. Approximately half of these patients were admitted to a subacute geriatrics or psychogeriatrics unit for ongoing care. Changed behaviours, including agitation/aggression and resistance to care, were common to all patients and did not determine whether patients were discharged from hospital by the acute geriatrics team or admitted to a subacute unit. However, factors, including younger age, male sex, independent mobility, previous specialist input for BPSD, higher psychotropic use and ‘code blacks’ in hospital, were associated with admission to a subacute unit (and a significantly longer length of stay), highlighting that patients with more severe BPSD that may be less responsive to non‐pharmacological strategies and carry a higher risk of harm to others are likely to require specialist inpatient care. Pajaro, To and Whitehead^3^ acknowledge their study's limitations, including its retrospective nature, small sample size and lack of BPSD severity measures. It also examined admissions of RACF residents only, when a recent prospective Australian study revealed that 50% of patients presenting with severe BPSD were from home and not RACFs.^4^ Nevertheless, it is one of only a handful to report on hospitalisation directly related to BPSD, and it compels us to consider how our hospitals meet (or do not meet) the needs of these patients and support the well‐being of the staff caring for them.

Best practice management of BPSD includes identifying and addressing physiological and psychological needs (such as fear, pain and hunger) and social and environmental triggers (such as unfamiliar surrounds and noise).^2^ However, most research has been conducted in the long‐term care setting and the applicability of these management principles to the acute care setting remains unclear.^1^ Management of BPSD in hospitals is particularly challenging due to busy ward conditions that are both over‐ and under‐stimulating and hard to modify, physical environments that are relatively fixed and lack dementia‐enabling design, rigid care routines that do not take personal preferences or diurnal rhythms into account, and limited staff skilled in dementia care and psychosocial interventions. Since clinicians may have limited ability to modify triggers and consistently provide effective non‐pharmacological interventions, they may find themselves relying upon chemical and/or physical restraint to address the risk of harm to the patient, co‐patients and staff, which in turn may trigger a cascade of negative sequelae and lead to poor outcomes for the patient.^1^

While clinicians working in this field recognise that there is great variability in the nature and severity of BPSD, which translates to a need for highly individualised risk assessment and management by specialist teams, the current approach to BPSD management in hospitals is overwhelmingly one of *dispersion*. Allocation of patients with BPSD to wards is generally ad hoc and piecemeal, determined by bed availability rather than capacity to deliver appropriate care. Patients are commonly allocated to single rooms or four‐bed close observation bays with no specific environmental modifications or staff with expertise in BPSD management. Research shows that the dispersed approach is inadequate, contributing to poor patient outcomes, overuse of restrictive practices and psychotropic medications, increased patient‐to‐staff violence, longer hospitalisations and high readmission rates.^1^ Hospitals must be encouraged to invest in models of care that can accommodate variation in BPSD severity and risk of harm to patients and staff.

One such model is the hospital‐based special care unit (SCU). Special care units incorporate secure built‐environments with dementia‐enabling designs, staffed by multidisciplinary teams specialised in geriatric medicine and/or psychogeriatrics. Special care units typically provide care for patients with moderate through to very severe BPSD. Our recent longitudinal study of an eight‐bed SCU in a tertiary hospital in Brisbane found that SCUs care was associated with a decrease in aggression severity, burden of neuropsychiatric symptoms and psychotropic use compared with standard medical ward care.^5^ Moreover, rehospitalisation rates for BPSD decreased following an SCU admission.^4^ Significant decreases in falls and occupational violence rates across the hospital's three medical wards and SCU ward also suggested a positive impact of this model of care on the overall care‐culture.^4^

With this emerging evidence base, we propose that hospitals should have an SCU for patients with severe BPSD. However, an SCU should be just one component of a tiered hospital‐wide approach to high quality and safe care for patients with BPSD. While many hospitals now offer rapid comprehensive geriatric assessment by a specialist team to facilitate early discharge of patients with BPSD back to the community, and more hospitals are opening SCUs for management of severe BPSD, we need to see greater investment in developing and implementing models of care for patients with BPSD who require more intensive psychosocial and physical resources than are currently available on standard hospital wards. At the very least, patients with BPSD and the staff who care for them stand to benefit from optimised ward environments and training and support to build workforce capability in delivering person‐centred dementia care. Benefits may then be realised by all older patients in hospital as the principles of excellent geriatric care extend across the acute care setting.^6^ Redesigning hospitals to meet the care needs of patients with dementia and BPSD demonstrates a commitment to age‐friendly, dignified care for all.

## CONFLICT OF INTEREST STATEMENT

There are no conflicts of interest to declare.

## References

[1] Anantapong K , Jiraphan A , Aunjitsakul W , et al. Behavioural and psychological symptoms of people with dementia in acute hospital settings: a systematic review and meta‐analysis. Age Ageing. 2025;54(1):afaf013. 39888603 10.1093/ageing/afaf013 PMC11784590

[2] Kales HC , Gitlin LN , Lyketsos CG . Assessment and management of behavioral and psychological symptoms of dementia. BMJ. 2015;350:h369. 25731881 10.1136/bmj.h369 PMC4707529

[3] Pajaro A , To T , Whitehead C . Factors associated with length of stay in patients from residential aged care facilities admitted for behavioural and psychological symptoms of dementia. Australas J Ageing. 2025;44(2):e70044. doi:10.1111/ajag.70044 40413752 PMC12103889

[4] Graham FA , Kelly L , Burmeister EA , et al. A hospital‐based special care unit for dementia decreased hospital readmission rates for behaviour while reducing rates of falls and occupational violence across medical wards. Age Ageing. 2025;54(4):afaf096. 40253685 10.1093/ageing/afaf096 PMC12009545

[5] Graham FA , Kelly L , Burmeister EA , et al. The impact of a hospital‐based special care unit on behavioural and psychological symptoms in older people living with dementia. Age Ageing. 2024;53(4):afae081. 38644744 10.1093/ageing/afae081

[6] Mudge A , Young A , McRae P , et al. Qualitative analysis of challenges and enablers to providing age friendly hospital care in an Australian health system. BMC Geriatr. 2021;21(1):147. 33639854 10.1186/s12877-021-02098-w PMC7913259

